# The Gibbs free energy of formation of halogenated benzenes, benzoates and phenols and their potential role as electron acceptors in anaerobic environments

**DOI:** 10.1007/s10532-014-9710-5

**Published:** 2014-09-18

**Authors:** Jan Dolfing, Igor Novak

**Affiliations:** 1 School of Civil Engineering and Geosciences, Newcastle University, Newcastle, NE1 7RU England, UK; 2 Faculty of Science, Charles Sturt University, Orange, NSW 2800 Australia

**Keywords:** Chlorophenol, Bromophenol, Dehalogenation, Redox potential, Chlorobenzoate, Chlorobenzene

## Abstract

The sequence of redox reactions in the natural environment generally follows the electron affinity of the electron acceptors present and can be rationalized by the redox potentials of the appropriate half-reactions. Answering the question how halogenated aromatics fit into this sequence requires information on their Gibbs free energy of formation values. In 1992 Gibbs free energy data for various classes of halogenated aromatic compounds were systematically explored for the first time based on Benson’s group contribution method. Since then more accurate quantum chemical calculation methods have become available. Here we use these methods to estimate enthalpy and Gibbs free energy of formation values of all chlorinated and brominated phenols. These data and similar state-of-the-art datasets for halogenated benzenes and benzoates were then used to calculate two-electron redox potentials of halogenated aromatics for standard conditions and for pH 7. The results underline the need to take speciation into consideration when evaluating redox potentials at pH 7 and highlight the fact that halogenated aromatics are excellent electron acceptors in aqueous environments.

**Electronic supplementary material:**

The online version of this article (doi:10.1007/s10532-014-9710-5) contains supplementary material, which is available to authorized users.

## Introduction

The seminal observations by Tiedje and co-workers on microbial dehalogenation have profoundly altered our perception of biodegradability of halogenated compounds (Suflita et al. 1982). We now know that anaerobic bacteria reductively dehalogenate a wide variety of organohalogens in a process called organohalide respiration, where organohalogens are used as electron acceptor by bacteria harnessing part of the energy released in the form of ATP (McCarty 1997; Farai et al. 2010; Leys et al. 2013). Based on this information treatment processes have been developed for the biodegradation of various classes of organohalogens including halophenols (Field and Sierra-Alvarez 2008). Insight in the microbial and thermodynamic logic behind the sequence of dehalogenation steps observed in these degradation processes (Dolfing 2003) requires an internally consistent set of data on the standard aqueous phase Gibbs free energy of formation (Δ*G*_f_^o^) and enthalpy of formation (Δ*H*_f_^o^) values for all congeners. For chlorophenols such a data set is currently not available. A previous set of data for chlorinated phenols (Dolfing and Harrison 1992) lacked values for Δ*H*_f_^o^, thus precluding its use at temperatures other than 298.15 K. Also, with the advent of accurate quantum chemical calculation methods and readily available computer power it is now possible to generate data sets that are more accurate than those generated 20 years ago. The objectives of the present paper are therefore (i) to present a state-of-the-art data set of Δ*G*_f_^o^ and Δ*H*_f_^o^ values for chlorinated phenols, and (ii) to do the same for brominated phenols. Speciation of halogenated phenols is pH dependent, and potentially affects the energetics of the dehalogenation reactions (Dolfing et al. 2010). Thus, our third objective is to outline the effect of pH on the change in Gibbs free energy for the reductive dehalogenation of halogenated phenols and on the redox potentials for the corresponding redox couples. In recent years Tang et al. (2010) and Sadowsky et al. (2013) have updated the existing database of thermochemical properties for halogenated aromatics with state-of-the-art quantum chemical information on chlorobenzoates and halobenzenes. The fourth objective of the present paper is to integrate these data into a consistent set of redox potentials for various classes of halogenated aromatics.

There is currently considerable interest in the use of microbial fuel cells for waste treatment, including waste containing halogenated phenols (Strycharz et al. 2010; Huang et al. 2012, 2014). Rational design and implementation of such systems hinges on precise knowledge of the amount of energy present in the waste (Heidrich et al. 2011). In addition to providing data for the prediction and rationalization of degradation pathways of various classes of halogenated aromatics in anaerobic environments (Dolfing 2003) the data presented here will allow a thermodynamic evaluation of the fraction of energy that is stored in the carbon-halogen bond of halogenated benzenes, benzoates and phenols.

## Materials and methods

### Computational methods

Ab initio quantum chemical calculations to estimate Δ*H*_f_^o^ and S^o^ values of phenol and all chlorinated and brominated phenol congeners were performed with the Gaussian 03 software, Revision E1 (Frisch et al. 2003). The use of this software for thermochemical calculations is well established (Novak 2004). The composite G3(MP2)/B3LYP method was used for calculation of total energies; the method (Baboul et al. 1999) has typical precision of 4 kJ/mol. The G3(MP2)/B3LYP method yields Δ*G*_f_^o^ and Δ*H*_f_^o^ values for the gas phase. For environmental applications data for the aqueous phase are generally more relevant. We therefore used the universal solvation model (Marenich et al. 2009) and G3(MP2)/B3LYP method to simulate water solvent as implemented in the Gaussian software to calculate Δ*G*_f_^o^ and Δ*H*_f_^o^ values for the aqueous solution.

### Gibbs free energy values

The standard molar Gibbs free energy of formation $\Delta G{\text{f}}^{o}_{\text{m}}$ was calculated from the equation $\Delta G_{\text{f}}^{o} = \Delta H_{\text{f}}^{o} - T[S^{o} {-} \varSigma \left( {\nu_{i} S_{i}^{o} } \right)]$ where Δ*H*_f_^*o*^ is the standard enthalpy of formation at 1 bar (100 kPa), *T* is the temperature of interest (298.15 K), *S* ^*o*^ is the absolute standard entropy, ν_*i*_ is the stoichiometric coefficient of element *I*, and *S*_*i*_^*ο*^ is the absolute entropy of element *I* in its standard reference state. *S*_*i*_^*ο*^ values used for carbon, hydrogen, oxygen, bromine and chlorine were 5.74, 65.34, 102.58, 76.11 and 111.54 J.K^−1^ mol^−1^ respectively (Cox et al. 1989). We have also calculated the appropriate total energies *H* ^*mol*^, *H* ^*at*^, *G* ^*mol*^, *G* ^*at*^ in the water solvent which allowed us to deduce the solvation energy correction for gas phase data and thus convert Δ*H*_f_^*o*^ (g) and Δ*G*_f_^*o o*^(g) values to Δ*H*_f_^*o*^ (l) and Δ*G*_f_^*o*^ (l). The exact expressions for *H* ^*mol*^, *H* ^*at*^, *G* ^*mol*^, *G* ^*at*^ are given in Gaussian G03 manual (Frisch et al. 2003).

The amount of free energy available from a reaction is given by the relationship $\Delta G^{\text{o}} = \varSigma \, \Delta G_{\text{f}}^{o} \left( {\text{products}} \right){-}\varSigma \, \Delta G_{\text{f}}^{o} \left( {\text{reactants}} \right)$ (Thauer et al. 1977). In aqueous solutions the standard state of all solutes is 1 mol/kg activity, that of water is the pure liquid. Under environmentally relevant conditions the concentrations of reactants and products are not 1 mol/kg. This is considered in Δ*G*′ values. For a hypothetical reaction *a*A + *b*B → *c*C + *d*D, Δ*G*′ values are calculated by using the mass equation

1 $$\Delta G^{\prime } {\mkern 1mu} = {\mkern 1mu} \Delta G^{o \prime} {\mkern 1mu} + {\mkern 1mu} RT{\mkern 1mu} \ln {\mkern 1mu} \left[ {\text{C}} \right]^{c} \left[ {\text{D}} \right]^{d} {\mkern 1mu} /{\mkern 1mu} \left[ {\text{A}} \right]^{a} \left[ {\text{B}} \right]^{b}$$

The Δ*G* ^*o*^′ value is obtained from the Δ*G* ^*o*^ value by making the appropriate corrections for pH 7 (Thauer et al. 1977). Δ*G*_f_^*o*^ values for inorganics were taken from Stumm and Morgan (1996).

For example for the hydrogen driven reductive dehalogenation of chlorobenzene to benzene, that is for chlorobenzene + H_2_ → benzene + H^+^ + Cl^−^:$\Delta G^{\text{o}} = \Delta G_{\text{f}}^{o} {\text{benzene}} + \Delta G_{\text{f}}^{o} {\text{H}}^{ + } + \Delta G_{\text{f}}^{o} {\text{Cl}}^{ - } {-}\Delta G_{\text{f}}^{o} {\text{chlorobenzene}} - \Delta G_{\text{f}}^{o} {\text{H}}_{ 2}$ and $\Delta G' = \Delta G^{o} + RT\,{ \ln }\left[ {\text{benzene}} \right]\left[ {{\text{H}}^{ + } } \right]\left[ {{\text{Cl}}^{ - } } \right]/\left[ {\text{chlorobenzene}} \right]\left[ {{\text{H}}_{ 2} } \right]$

### Speciation and pH

Halophenols are weak acids, but stronger than phenol. In waste water weak acids are partially ionized and are in thermodynamic equilibrium with their conjugate bases. The notion that these species are in equilibrium implies that Gibbs free energy values for reactions where these compounds are reactants or products are calculated by using the Δ*G*_f_ values of either the acid, with the formula

2 $$\Delta G_{\text{f}} = \Delta G_{\text{f}}^{{{\text{o}} }} \, + {\text{RT ln}}\,\alpha$$

or the conjugated base with the formula

3 $$\Delta G_{\text{f}} = \Delta G_{\text{f}}^{\text{o}} + {\text{ RT ln }}(1 - \alpha )$$

where $\alpha = 10^{{ - {\text{pH}}}} /\left( { 10^{{ - {\text{pH}}}} + 10^{{ - {\text{p}}K{\text{a}}}} } \right)$ (Dolfing et al. 2010). Δ*G*_f_ values for halobenzoates were calculated after Dolfing and Harrison (1992) as

4 $$\Delta G_{\text{f}} {\text{halobenzoate}} = \Delta G_{\text{f}} {\text{halobenzoic acid}} + 2.3{\text{RTp}}K_{\text{a}}$$

### Gibbs free energies of chlorinated benzenes

Gibbs free energy of formation data for chlorinated benzenes in the aqueous phase (in kcal mol^−1^) were taken from Sadowsky et al. (2013) and converted to kJ mol^−1^ (1 kcal = 4.184 kJ). These values were used to calculate the change in Gibbs free energy for the reductive dehalogenation reactions as described previously (Dolfing and Harrison 1992).

### Redox potentials

Two electron reduction potentials were calculated after Thauer et al. (1977). For example: based on *G*_f_^*o*^ values of −138.5 and −133.5 kJ mol^−1^ for C_6_Cl_6_ and C_6_Cl_5_H respectively and values of 0 and −39.95 for H^+^ at pH 0 and pH 7 respectively, and with Δ*G*_f_ H_2_ (gas) = 0 kJ mol^−1^; Δ*G*_f_ Cl^−^ = −131.3 kJ mol^−1^ (Stumm and Morgan 1996) reductive dechlorination of hexachlorobenzene to pentachlorobenzene according to C_6_Cl_6_ + H_2_ (gas) → C_6_Cl_5_H + H^+^ + Cl^−^ yields −126.3 kJ mol^−1^ under standard conditions (pH 0) and −166.2 kJ mol^−1^ at pH 7. To calculate the corresponding redox potentials these values are then divided by −nF/1,000,000 where *n* is the number of electrons transferred in the reaction and F is the Faraday constant (96485 J/V) (Stumm and Morgan 1996) and 1,000,000 is the multiplication factor to account for conversion of kJ to mV rather than J to V. This would yield reduction potentials of 654 and 861 mV for pH 0 and pH 7 respectively. For pH 7 the latter value still needs to be corrected for the redox potential of the H^+^/H_2_ redox couple, which is −414 mV at pH 7 (and indeed 0 at pH 0). Thus the redox potentials of the C_6_Cl_6_/C_6_Cl_5_H redox couple are 654 mV at pH 0 and 447 mV at pH 7 respectively.

## Results and discussion

### Δ*G*_f_^o^ and Δ*H*_f_^o^ values of chlorinated phenols

Quantum mechanical methods discriminate between conformers that are deemed to represent the same compound in the environment. For example, Δ*G*_f_^o^ for syn-2-chlorophenol (2-chlorophenol) differs from Δ*G*_f_^o^ for anti-2-chlorophenol (6-chlorophenol) (Supporting Information (SI) Table S1). This difference reflects the presence or absence of intramolecular hydrogen interaction (“bond”) between hydroxyl hydrogen and the halogen. In environmental chemistry this distinction between conformers is not made, because in the environment each congener is present in the conformation that has the lowest energy. In the supporting material Δ*G*_f_^o^ and Δ*H*_f_^o^ values for all 31 chlorophenol congeners are provided. In Table 1 we present Δ*G*_f_^o^ and Δ*H*_f_^o^ values for the environmentally relevant congeners.

**Table 1:** Thermodynamic data for chlorinated phenols under standard conditions (in kJ mol^−1^ **)** ^a^

	Δ*H*_f_^o^_gas_	Δ*G*_f_^o^_gas_	Δ*H*_f_^o^_aq_	Δ*G*_f_^o^_aq_	Δ*H*_f_^o^_gas(exp)_^b^
Phenol	−96.4	−51.2	−118.0	−72.8	−96.4
2-chlorophenol	−132.8	−83.7	−143.9	−95.2	
3-chlorophenol	−126.3	−77.8	−148.4	−99.9	
4-chlorophenol	−124.2	−75.7	−147.0	−98.6	
2,3-dichlorophenol	−154.7	−101.7	−165.1	−112.4	−151.6
2,4-dichlorophenol	−156.6	−104.2	−168.1	−116.3	−156.3
2,5-dichlorophenol	−162.2	−109.7	−173.1	−120.9	−158.4
2,6-dichlorophenol	−152.6	−99.9	−164.7	−112.8	−146.3
3,4-dichlorophenol	−145.9	−93.7	−168.3	−116.3	−150.3
3,5-dichlorophenol	−151.8	−99.9	−173.0	−121.2	−148.2
2,3,4-trichlorophenol	−174.8	−118.1	−184.3	−128.0	
2,3,5-trichlorophenol	−179.6	−123.3	−188.5	−132.3	
2,3,6-trichlorophenol	−173.9	−117.4	−184.4	−131.0	
2,4,5-dichlorophenol	−177.3	−121.1	−187.6	−131.8	
2,4,6-trichlorophenol	−180.5	−124.5	−191.3	−136.5	
3,4,5-trichlorophenol	−167.2	−111.2	−188.1	−132.0	
2,3,4,5-tetrachlorophenol	−191.7	−131.4	−199.6	−139.4	
2,3,4,6-tetrachlorophenol	−192.4	−132.2	−200.5	−140.0	
2,3,5,6-tetrachlorophenol	−193.8	−133.5	−201.4	−142.2	
Pentachlorophenol	−202.5	−137.9	−207.9	−144.3	

^a^Standard conditions are: 25 °C; 100 kPa (gas phase) or 1 M (aqueous solution)

^b^Experimental values are from Linstrom and Mallard (2012)

**Fig. 1:**
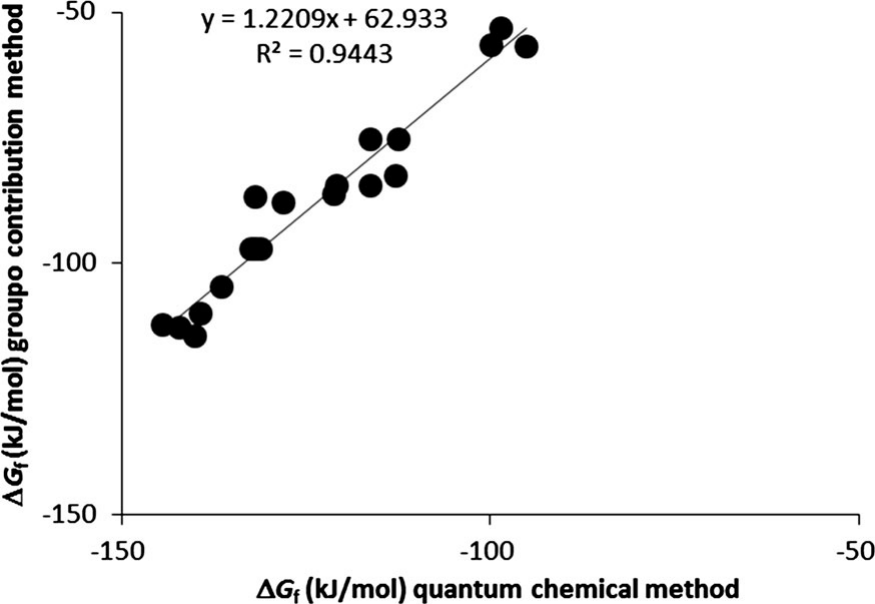
Gibbs free energy of formation values of chlorinated phenols in the aqueous phase (1 M; 25 °C). Values obtained with a group contribution method are from Dolfing and Harrison (1992)

**Fig. 2:**
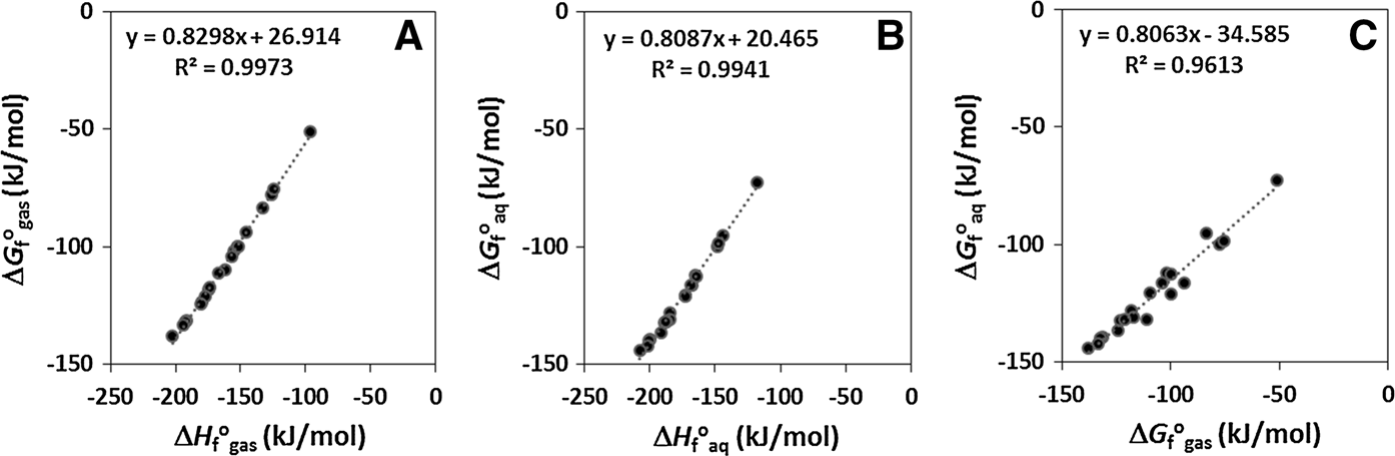
Relationships between thermodynamic parameters of chlorophenols. **a** Relationship between Δ*G*_f_^o^_gas_ and Δ*H*_f_^o^_gas_, **b** relationship between Δ*G*_f_^o^_aq_ and Δ*H*_f_^o^_aq_, and **c** relationship between Δ*G*_f_^o^_aq_ and Δ*G*_f_^o^_gas_

Table 1 shows the Δ*H*_f_^o^ and Δ*G*_f_^o^ values for all 19 environmentally relevant chlorophenols for both the gaseous and the aqueous phase. The Δ*G*_f_^o^ values range between −75.7 and −137.9 kJ/mol for the gas phase and between −95.2 and −144.3 kJ/mol for the aqueous phase. These values are lower than those previously reported (Dolfing and Harrison 1992). There is considerable scatter in plots of the new versus these “old” data (Fig. 1). This is not surprising since quantum mechanical methods incorporate interactions that are not taken into account by group contribution methods which rely on transferability and averaging of properties of a particular functional group. The correlations between Δ*H*_f_^o^_gas_ and Δ*G*_f_^o^_gas_, and between Δ*H*_f_^o^_aq_ and Δ*G*_f_^o^_aq_ (Fig. 2) are excellent, while the correlation between Δ*G*_f_^o^_gas_ and Δ*G*_f_^o^_aq_ (Fig. 2) is less perfect, which reflects *inter alia* the influence of molecular structure on solvent solute interactions.

### Δ*G*_f_^o^ and Δ*H*_f_^o^ values of brominated phenols

**Fig. 3:**
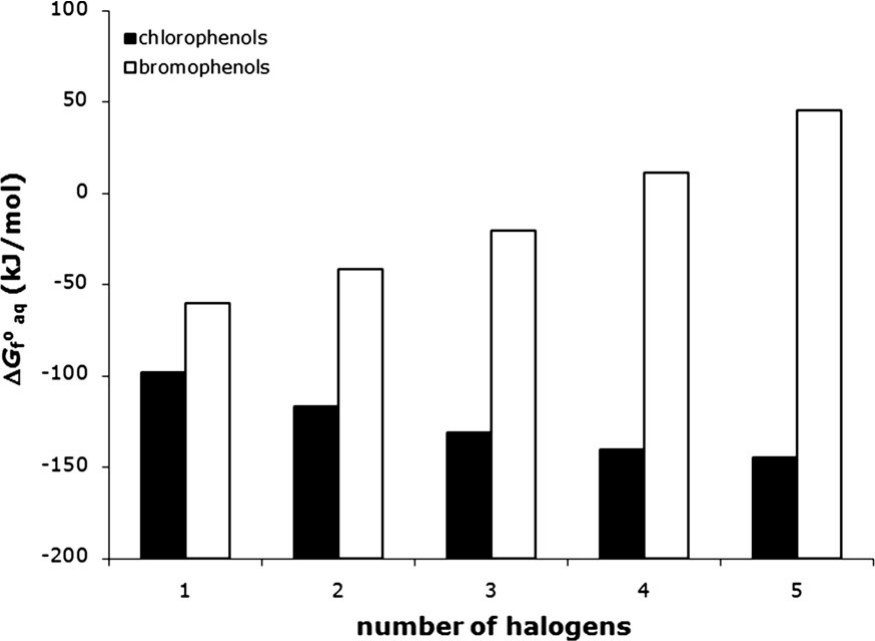
Effect of the number of halogen substituents on the Gibbs free energy of formation of chloro- and bromophenols

**Fig. 4:**
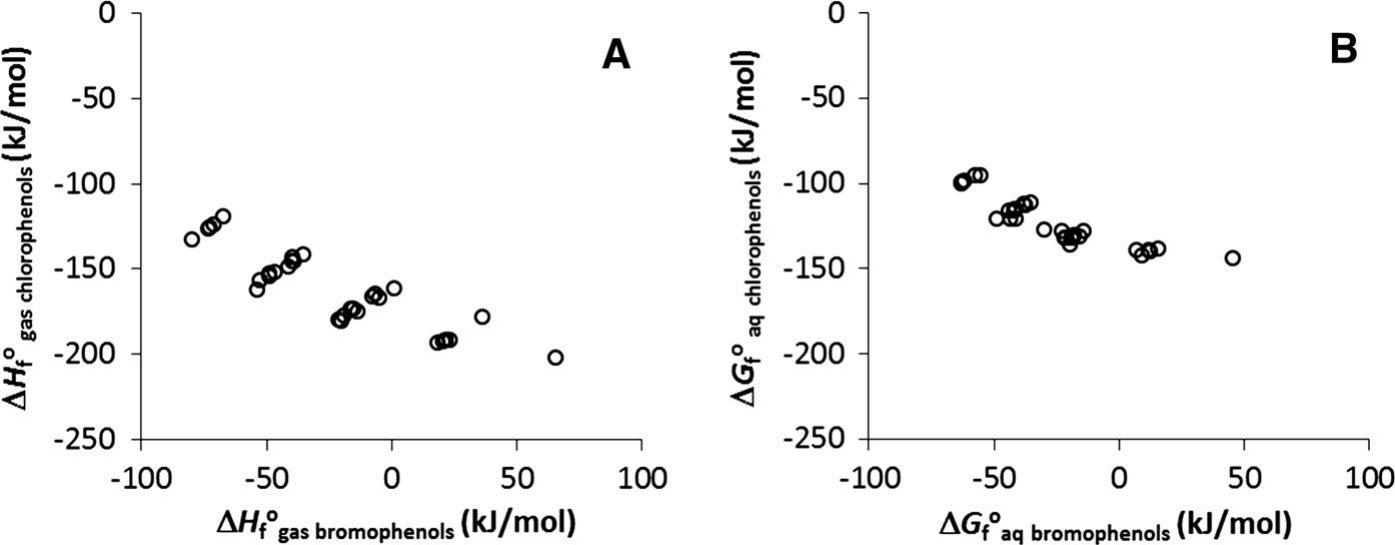
Correlation between thermodynamic parameters of chlorophenols and bromophenols; **a** Δ*H*_f_^o^_gas_, and **b** Δ*G*_f_^o^_aq_

Table 2 shows the Δ*H*_f_^o^ and Δ*G*_f_^o^ values for all 19 environmentally relevant bromophenols for both the gas and the aqueous phase (the data for the full series of 31 congeners is provided in SI Table S2. The Δ*G*_f_^o^ values range between −45.1 and 59.1 kJ/mol for the gas phase and between −63.0 and 45.7 kJ/mol for the aqueous phase. Contrary to the case for chlorophenols Δ*G*_f_^o^_aq_ values for bromophenols decrease with increasing degree of halogenation (Fig. 3). Plots of Δ*H*_f_^o^_gas_ and Δ*G*_f_^o^_aq_ values of chlorinated phenols versus those of bromophenols illustrate that the effect of chloro substituents on the stability of compound is fundamentally different from that of bromo substituents (Fig. 4). This is due to the fact that chlorine is a more electronegative element than bromine, and because bromine is a larger atom, which will introduce steric repulsion (and hence destabilization) with neighboring substituents (be these substituents hydrogens, bromines or OH groups).

**Table 2:** Thermodynamic data for brominated phenols under standard conditions (in kJ mol^−1^)^a^

	Δ*H*_f_^o^_gas_	Δ*G*_f_^o^_gas_	Δ*H*_f_^o^_aq_	Δ*G*_f_^o^_aq_	Δ*H*_f_^o^_gas(exp)_^b^
Phenol	−96.4	−51.2	−118.0	−72.8	−96.4
2-bromophenol	−79.9	−45.1	−91.8	−57.3	
3-bromophenol	−72.9	−38.6	−97.4	−63.0	
4-bromophenol	−71.0	−37.0	−96.0	−62.0	
2,3-dibromophenol	48.7	24.3	−62.0	−37.9	
2,4-dibromophenol	52.5	28.7	−66.9	−43.6	
2,5-dibromophenol	53.7	29.9	−67.3	−43.3	
2,6-dibromophenol	−49.0	−24.9	−60.6	−37.6	
3,4-dibromophenol	−39.4	−15.8	−65.6	−42.0	
3,5-dibromophenol	−46.7	−23.5	−72.1	−49.0	
2,3,4-tribromophenol	−13.3	0.1	−42.5	−29.7	
2,3,5-tribromophenol	−21.2	−7.7	−34.6	−21.2	
2,3,6-tribromophenol	−16.3	−2.7	−27.9	−15.4	
2,4,5-tribromophenol	−18.9	−5.6	−34.0	−21.6	
2,4,6-tribromophenol	−19.8	−6.9	−30.9	−19.6	−0.9
3,4,5-tribromophenol	−4.7	8.5	−32.0	−19.0	
2,3,4,5-tetrabromophenol	23.4	26.8	8.2	12.0	
2,3,4,6-tetrabromophenol	20.5	23.7	9.9	7.3	
2,3,5,6-tetrabromophenol	18.7	21.9	8.1	9.3	
Pentabromophenol	65.6	59.1	54.8	45.7	

^a^Standard conditions are: 25 °C; 100 kPa (gas phase) or 1 M (aqueous solution)

^b^Experimental values are from Linstrom and Mallard (2012)

### Reliability of calculated standard enthalpy of formation values of halophenols

The experimental standard enthalpies of formation in the gas phase for some halophenols and the parent phenol (Linstrom and Mallard 2012) were used to assess the reliability of our calculated values (Tables 1–2). We note that that agreement with experimental and calculated values for most chlorophenols is close to the stated uncertainty of 4 kJ/mol. However, the discrepancy between calculated and experimental standard enthalpy for 2,4,6-tribromophenol (the only one for which Δ*H*_f_^o^ had been reported) is much larger and suggests that the measured value (Linstrom and Mallard 2012) needs to be reassessed.

### Halogenated phenols, speciation and pH

pH affects the speciation of halophenols (Mun et al. 2008). Dissociation of a halophenol results in the formation of a halophenolate and hence a decrease in the concentration of the halophenol. The degree of dissociation depends on the pH and the p*K*_*a*_ value of the halophenol congener (Dolfing et al. 2010). Table 3 (chlorophenols) and SI Table 3 (bromophenols) list the Δ*G*_f_^o^ values corrected for dissociation at pH 7 for chlorophenols and bromophenols, illustrating that the effect of dissociation is not necessarily negligible. This has implications for the energetics of the dehalogenation reaction. The p*K*_a_ increases with decreasing degree of halogenation; this implies that deprotonation has a stabilizing effect on highly halogenated compounds. *Ortho* halophenols are more acidic than *meta* and *para* halophenols because of the large inductive effect of the halogen on the vicinal hydroxyl group (Han et al. 2004). For the same reason the acidity of halophenols increases with the number of halogen substitutions (Table 3 and SI Table S3).

**Table 3:** Effect of p*K*_*a*_ on speciation and Δ*G*_f_^o^_aq_ at pH 7 for chlorophenols^a^

		Δ*G*_f_^o^_aq_	p*K*_*a*_	α^b^	Δ*G*_f aq_^o′^ ^c^
Phenol		−72.8	10.00	1.00	−72.8
2-chlorophenol		−95.2	8.46	0.97	−95.3
3-chlorophenol		−99.9	8.92	0.99	−99.9
4-chlorophenol		−98.6	9.13	0.99	−98.6
2,3-dichlorophenol		−112.4	7.90	0.89	−112.7
2,4-dichlorophenol		−116.3	7.94	0.90	−116.5
2,5-dichlorophenol		−120.9	7.35	0.69	−121.8
2,6-dichlorophenol		−112.8	6.49	0.24	−116.3
3,4-dichlorophenol		−116.3	8.43	0.96	−116.4
3,5-dichlorophenol		−121.2	7.87	0.88	−121.5
2,3,4-trichlorophenol		−128.0	7.53	0.77	−128.7
2,3,5-trichlorophenol		−132.3	6.79	0.38	−134.7
2,3,6-trichlorophenol		−131.0	5.65	0.04	−138.8
2,4,5-dichlorophenol		−131.8	6.90	0.44	−133.8
2,4,6-trichlorophenol		−136.5	5.78	0.06	−143.6
3,4,5-trichlorophenol		−132.0	7.39	0.71	−132.8
2,3,4,5-tetrachlorophenol		−139.4	6.63	0.30	−142.4
2,3,4,6-tetrachlorophenol		−140.0	5.11	0.01	−150.8
2,3,5,6-tetrachlorophenol		−142.2	5.05	0.01	−153.3
Pentachlorophenol		−144.3	4.84	0.01	−156.7

^a^p*K*_*a*_ values are taken from Han and Tao (2006); Δ*G*_f_^o^_aq_ and $\Updelta G_{{\rm f}\, {\rm aq}}^{o\prime}$ are in kJ mol^−1^

^b^α is the fraction of chlorinated phenol present as chlorophenols; the fraction present as chlorophenolate is 1−α

^c^Δ*G*_f_^o^ *’*_aq:_ $$\Updelta G_{{\rm f}\, {\rm aq}}^{o\prime}$$ at pH 7

### Redox potentials of halogenated phenols

**Fig. 5:**
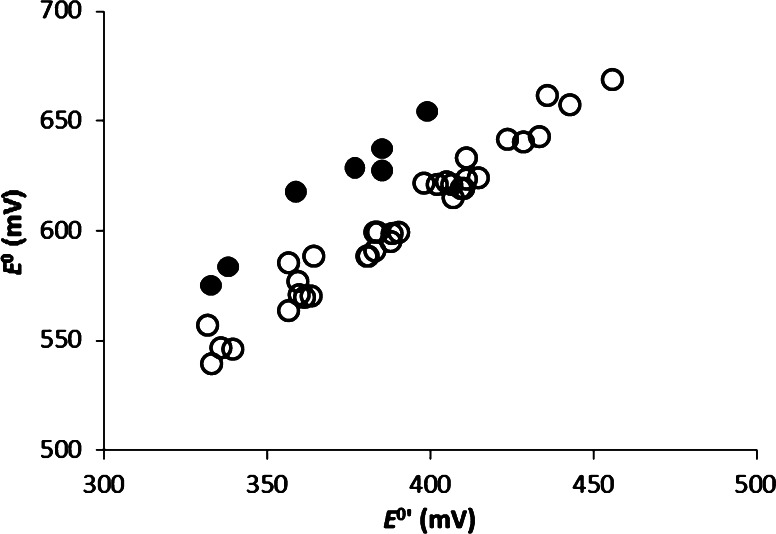
Redox potentials for reductive dechlorination of chlorinated phenols under standard conditions (*E* ^0^) versus the redox potentials for the same redox couple at pH 7 (*E* ^0^′). *Black dots* indicate redox couples representing ortho dechlorination of double ortho flanked hydroxyl groups

With H_2_ as electron donor reductive dehalogenation of halophenols is an exergonic process. Under standard conditions the change in Gibbs free energy values for reductive dehalogenation of chlorophenols and bromophenols are in the range of −104 to −129 kJ per mol chloride released and of −112 to −146 kJ per mol bromide released respectively. At pH 7 reductive dehalogenation is significantly more favorable than at pH 0 because protons are generated as reaction product, and because with increasing pH an increasing fraction of the phenols is deprotonated. The two electron reduction potentials naturally follow this drift (Table 4 for chlorophenols; SI Table S4 for bromophenols). A plot of the redox potentials of all redox couples for chlorinated phenols at pH 7 versus the corresponding redox potentials under standard conditions illustrates that compared to pH 0 pH 7 especially favors *meta* and *para* dechlorination over *ortho* dechlorination (Fig. 5). A similar *ortho* effect was not observed for brominated phenols (data not shown).

**Table 4:** Gibbs free energy values and redox potentials for the reductive dechlorination of chlorophenols with H_2_ (gas) as electron donor^a^

Reactant	Product	Δ*G* ^o^	Δ*G* ^o^′	*E* ^o^	*E* ^o^′
Pentachlorophenol	2,3,4,5-tetrachlorophenol	−126.3	−157.0	655	399
	2,3,4,6-tetrachlorophenol	−127.0	−165.4	658	443
	2,3,5,6-tetrachlorophenol	−129.2	−167.9	669	456
2,3,4,5-tetrachlorophenol	2,3,4-trichlorophenol	−120.0	−157.6	622	402
	2,3,5-trichlorophenol	−124.3	−163.6	644	434
	2,4,5-trichlorophenol	−123.7	−162.7	641	429
	3,4,5-trichlorophenol	−123.9	−161.7	642	424
2,3,4,6-tetrachlorophenol	2,3,4-trichlorophenol	−119.3	−149.1	618	358
	2,3,6-trichlorophenol	−122.3	−159.2	634	411
	2,4,5-trichlorophenol	−123.0	−154.2	638	385
	2,4,6-trichlorophenol	−127.8	−164.0	662	436
2,3,5,6-tetrachlorophenol	2,3,5-trichlorophenol	−121.5	−152.7	629	377
	2,3,6-trichlorophenol	−120.1	−156.7	622	398
2,3,4-trichlorophenol	2,3-dichlorophenol	−115.7	−155.3	599	391
	2,4-dichlorophenol	−119.5	−159.1	619	410
	3,4-dichlorophenol	−119.6	−159.0	619	410
2,3,5-trichlorophenol	2,3-dichlorophenol	−111.4	−149.2	577	359
	2,5-dichlorophenol	−119.9	−158.4	621	407
	3,5-dichlorophenol	−120.1	−158.0	622	405
2,3,6-trichlorophenol	2,3-dichlorophenol	−112.7	−145.1	584	338
	2,5-dichlorophenol	−121.2	−154.3	628	385
	2,6-dichlorophenol	−113.1	−148.8	586	357
2,4,5-trichlorophenol	2,4-dichlorophenol	−115.8	−154.0	600	384
	2,5-dichlorophenol	−120.4	−159.3	624	411
	3,4-dichlorophenol	−115.8	−153.9	600	383
2,4,6-trichlorophenol	2,4-dichlorophenol	−111.1	−144.2	575	333
	2,6-dichlorophenol	−107.6	−144.0	557	332
3,4,5-trichlorophenol	3,4-dichlorophenol	−115.6	−154.8	599	388
	3,5-dichlorophenol	−120.5	−159.9	624	415
2,3-dichlorophenol	2-chlorophenol	−114.1	−153.9	591	383
	3-chlorophenol	−118.8	−158.5	615	407
2,4-dichlorophenol	2-chlorophenol	−110.2	−150.0	571	363
	4-chlorophenol	−113.7	−153.4	589	381
2,5-dichlorophenol	2-chlorophenol	−105.6	−144.7	547	336
	3-chlorophenol	−110.2	−149.3	571	360
2,6-dichlorophenol	2-chlorophenol	−113.7	−150.2	589	364
3,4-dichlorophenol	3-chlorophenol	−114.9	−154.8	595	388
	4-chlorophenol	−113.7	−153.5	589	382
3,5-dichlorophenol	3-chlorophenol	−110.0	−149.7	570	362
2-chlorophenol	Phenol	−108.9	−148.8	564	357
3-chlorophenol	Phenol	−104.3	−144.2	540	333
4-chlorophenol	Phenol	−105.5	−145.4	546	339

^a^Standard conditions are 25 °C; solutes at 1 M, H_2_ gas at 1 atm; Δ*G* ^o^ *′* and *E* ^o^′ are for pH 7. Δ*G* values are in kJ reaction^−1^; *E* values are in mV

### Redox potentials of chlorinated benzoates

**Fig. 6:**
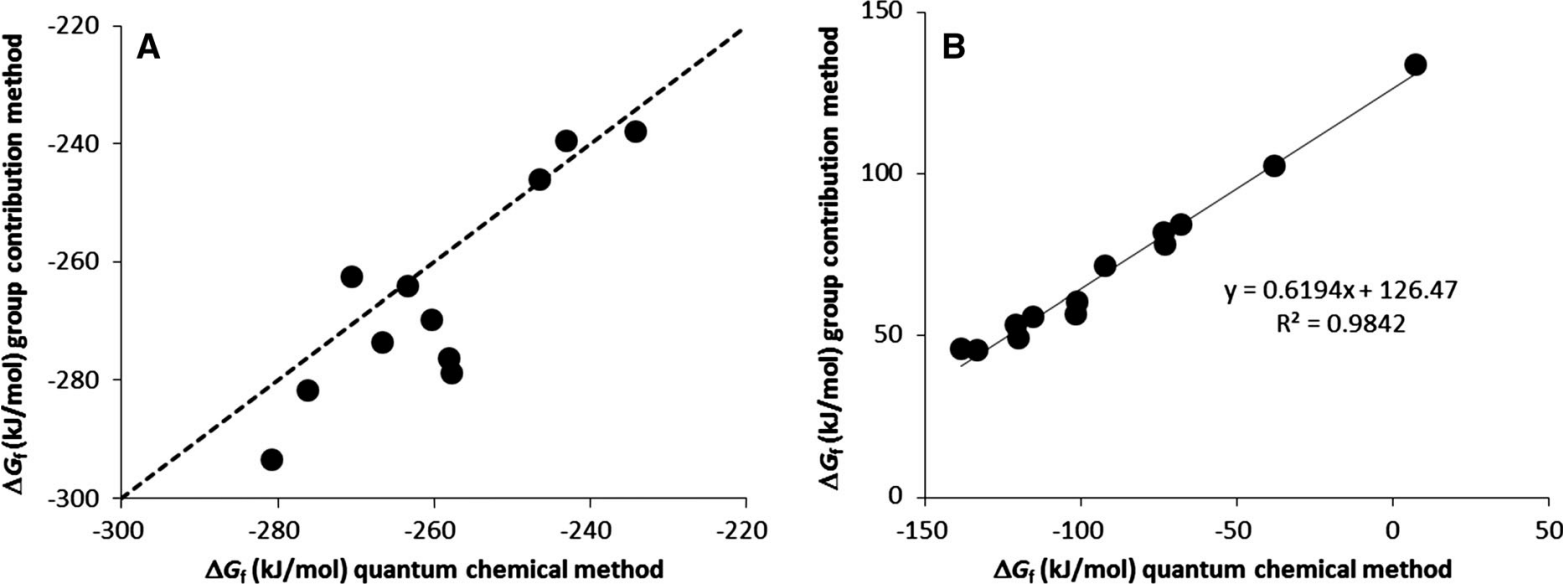
Gibbs free energy of formation values of **a** chlorinated benzoates and **b** chlorinated benzenes in the aqueous phase (1 M; 25 °C). Values obtained with quantum chemical methods are based on Tang et al. (2010) and on Sadowsky et al. (2013) respectively; values obtained with group contribution methods are from Dolfing and Harrison (1992). The *dotted line* in **a** is the 1:1 line; a *trendline* in **a** (not shown) would have *R* ^2^ = 0.76

Chlorinated benzoates are the compounds with which Tiedje and co-workers made their seminal observations on microbial dehalogenation (Suflita et al. 1982). (Tang et al. 2010) recently used quantum chemical methods (at the G3XMP2 level) plus a polarizable conductor model to estimate Gibbs free energy of formation values of chlorinated benzoic acids for both the gas and the aqueous phase. At pH 7 chlorinated benzoic acids are essentially fully deprotonated: their p*K*_*a*_ values range between −3.3 and 3.6 (Tang et al. 2010). Table 5 lists the Gibbs free energy values of all 19 chlorobenzoate congeners. A plot of these values versus those obtained with Benson’s group contribution method (Dolfing and Harrison 1992) reveals a less than perfect correlation (Fig. 6a) indicating that analogous to the case for halophenols (Fig. 1) quantum chemical methods incorporate electronic interactions that are not taken into account by group contribution methods. The two electron reduction potentials for chlorobenzoic acids (Table 6) range between 560 and 707 mV. The redox potentials for chlorobenzoates at pH 7 range between 285 and 501 mV. These values are systematically different from those reported previously by Tang et al. (2010) who neglected to make the appropriate correction for the H^+^/H_2_ redox couple at pH 7.

**Table 5:** Δ*G*_f_^o^_aq_ (in kJ mol^−1^) for chlorobenzoates

	Benson’s method^a^	Quantum chemical method^b^
2-chlorobenzoate	−237.9	−234.2
3-chlorobenzoate	−246.0	−246.5
4-chlorobenzoate	−239.5	−243.0
2,3-dichlorobenzoate	−269.7	−260.4
2,4-dichlorobenzoate	−276.4	−258.0
2,5-dichlorobenzoate	−287.7	−257.8
2,6-dichlorobenzoate	−262.6	−270.5
3,4-dichlorobenzoate	−264.2	−263.3
3,5-dichlorobenzoate	−273.5	−266.6
2,3,4-trichlorobenzoate		−273.4
2,3,5-trichlorobenzoate	−293.4	−280.7
2,3,6-trichlorobenzoate		−287.4
2,4,5-dichlorobenzoate		−271.0
2,4,6-trichlorobenzoate		−286.6
3,4,5-trichlorobenzoate	−281.6	−276.1
2,3,4,5-tetrachlorobenzoate		−275.3
2,3,4,6-tetrachlorobenzoate		−296.8
2,3,5,6-tetrachlorobenzoate		−296.0
Pentachlorobenzoate		−299.6

^a^Values taken from Dolfing and Harrison (1992)

^b^Calculated from values in Tang et al. (2010) using Eq. 4

**Table 6:** Gibbs free energy values and redox potentials for the reductive dechlorination of chlorobenzoic acids (at pH 0) and chlorobenzoates (at pH 7) with H_2_ (gas) as electron donor^a^

Reactant	Product	Δ*G* ^o^	Δ*G* ^o^′	*E* ^o^	*E* ^o^′
Pentachloro	2,3,4,5-tetrachloro	−129.6	−146.8	672	347
	2,3,4,6-tetrachlorol	−128.7	−168.4	667	458
	2,3,5,6-tetrachlorol	−126.9	-167.5	658	454
2,3,4,5-tetrachloro	2,3,4-trichloro	−124.7	-169.3	646	463
	3,4,5-trichloro	−128.0	-176.6	663	501
	2,4,5-trichloro	−128.0	-166.9	663	451
	2,3,5-trichloro	−136.5	-172.0	707	477
2,3,4,6-tetrachloro	2,3,4-trichloro	−125.6	-147.7	651	352
	2,3,6-trichloro	−124.7	-161.7	646	424
	2,4,5-trichloro	−128.9	−145.3	668	339
	2,4,6-trichloro	−126.2	−160.9	654	420
2,3,5,6-tetrachloro	2,3,5-trichloro	−130.7	−155.9	677	394
	2,3,6-trichloro	−126.5	−162.6	656	428
2,3,4-trichloro	2,3-dichloro	−121.5	−158.1	630	405
	2,4-dichloro	−125.1	−155.8	648	393
	3,4-dichloro	−135.5	−161.1	702	421
2,3,5-trichloro	2,3-dichloro	−118.2	−150.7	613	367
	3,5-dichloro	−122.3	−148.2	634	354
	2,5-dichloro	−133.3	−157.0	691	400
2,3,6-trichloro	2,3-dichloro	−122.4	−144.1	634	333
	2,6-dichloro	−126.5	−141.5	656	319
	2,5-dichloro	−119.9	−154.2	621	385
2,4,5-trichloro	2,4-dichloro	−121.8	−158.2	631	406
	2,5-dichloro	−122.3	−157.9	634	404
	3,4-dichloro	−132.2	−163.5	685	433
2,4,6-trichloro	2,4-dichloro	−124.5	−142.6	645	325
	2,6-dichloro	−118.4	−155.0	614	389
3,4,5-trichloro	3,4-dichloro	−123.7	−158.3	641	407
	3,5-dichloro	−124.8	−161.6	647	424
2,3-dichloro	2-chloro	−118.3	−145.1	613	338
	3-chloro	−131.9	−157.4	684	401
2,4-dichloro	2-chloro	−114.7	−147.4	594	350
	4-chloro	−127.2	−156.1	659	395
2,5-dichloro	2-chloro	−114.2	−147.6	592	351
	3-chloro	−127.8	−159.9	662	415
2,6-dichloro	2-chloro	−120.8	−135.0	626	285
3,4-dichloro	3-chloro	−117.9	−154.4	611	386
	4-chloro	−116.8	−150.8	605	368
3,5-dichloro	3-chloro	−116.8	−151.1	605	369
2-chloro	Benzoic acid/benzoate	−121.6	−154.1	630	385
3-chloro	Benzoic acid/benzoate	−108.0	−141.8	560	321
4-chloro	Benzoic acid/benzoate	−109.1	−145.4	565	339

^a^Standard conditions are 25 °C; solutes at 1 M, H_2_ gas at 1 atm; Δ*G* ^o^ and *E* ^o^ are for pH 0; $\Updelta G^{o\prime}$ and *E* ^o^′ are for pH 7. Δ*G* values are in kJ reaction^−1^; *E* values are in mV. Values are based on Tang et al. (2010) with corrections for pH 7 calculated using Eq. 4

### Redox potentials of halogenated benzenes

Sadowsky et al. (2013) recently used quantum chemical methods at the 6−311+G(3df,2p) level plus the SMD implicit solvation model to estimate thermochemical properties of (poly)halobenzenes. A plot of the aqueous Gibbs free energy of formation values of chlorinated benzenes as obtained with Benson’s group contribution method (Dolfing and Harrison 1992) versus the values obtained by Sadowsky et al. (2013) (Fig. 6b) shows a reasonably good correlation between the two approaches. A large part of the discrepancy between the two data sets appears due to the estimate for benzene itself, without any substituents. The scatter seems less than for the analogous comparison for chlorinated benzenes and benzoates (cf Fig. 1 and Fig. 6a), suggesting that one of the major weaknesses of the group contribution method was the lack of detail of important interactions between the hydroxyl group and the halogen substituents in the case of the chlorophenols and between the carboxyl group and the halogen substituents in the case of the chlorobenzoates. The redox potentials for polyhalogenated benzenes range between 446 and 654 mV at pH 0 and between 239 and 447 mV at pH 7 respectively (Table 7). The latter values are considerably lower than those listed by Sadowsky et al. (2013).

**Table 7:** Gibbs free energy values and redox potentials for the reductive dechlorination of chlorinated benzenes with H_2_ (gas) as electron donor^a^

Reactant	Product	Δ*G* ^0^	Δ*G* ^0′^	*E* ^0^	*E* ^0′^
Hexachlorobenzene	Pentachlorobenzene	−126.3	−166.2	654	447
Pentachlorobenzene	1,2,3,4-tetrachlorobenzene	−113.3	−153.3	587	380
	1,2,3,5-tetrachlorobenzene	−117.9	−157.9	611	404
	1,2,4,5-tetrachlorobenzene	−118.7	−158.7	615	408
1,2,3,4-tetrachlorobenzene	1,2,3-trichlorobenzene	−108.3	−148.2	561	354
	1,2,4-trichlorobenzene	−117.1	−157.0	607	400
1,2,3,5-tetrachlorobenzene	1,2,3-trichlorobenzene	−103.7	−143.6	537	330
	1,3,5-trichlorobenzene	−112.9	−152.8	585	378
	1,2,4,-trichlorobenzene	−112.5	−152.4	583	376
1,2,4,5-tetrachlorobenzene	1,2,4-trichlorobenzene	−111.6	−151.6	579	372
1,2,3-trichlorobenzene	1,2-dichlorobenzene	−106.6	−146.6	552	346
	1,3-dichlorobenzene	−112.5	−152.4	583	376
1,2,4-trichlorobenzene	1,2-dichlorobenzene	−97.8	−137.8	507	300
	1,3-dichlorobenzene	−103.7	−143.6	537	330
	1,4-dichlorobenzene	−103.3	−143.2	535	328
1,3,5-trichlorobenzene	1,3-dichlorobenzene	−103.3	−143.2	535	328
1,2-dichlorobenzene	Monochlorobenzene	−101.6	−141.5	526	320
1,3-dichlorobenzene	Monochlorobenzene	−95.7	−135.7	496	289
l,4-dichlorobenzene	Monochlorobenzene	−96.2	−136.1	498	291
Monochlorobenzene	Benzene	−86.1	−126.1	446	239

^a^Standard conditions are 25 °C; solutes at 1 M, H_2_ gas at 1 atm; Δ*G* ^o^ and *E* ^o^ are for pH 0; $\Updelta G^{o\prime}$ and *E* ^o^′ are for pH 7. Δ*G* values are in kJ reaction^−1^; *E* values are in mV. Values are based on Gibbs free energies in (Sadowsky et al. 2013); see Materials and methods for details

### Quantum chemical methods versus Benson’s group contribution method

A comparison of Gibbs free energy of formation values and redox potentials for various classes of halogenated aromatics obtained with Benson’s method versus datasets obtained with quantum chemical methods illustrates that quantum chemical methods allow a level of precision not achievable with group contribution methods. Not only was there a less than perfect agreement between the respective datasets for chlorobenzoates and chlorophenols there was also considerable scatter (Fig. 1 and Fig. 6a). Interestingly, this scatter was far less for the chlorinated benzenes (Fig. 6b). Thus it appears that the group contribution method did especially poor for interactions between the carboxy and the hydroxy group on the one hand and the chloro substituents on the other hand. Another interesting observation is that the consensus Gibbs free energy value for benzene in the aqueous phase in the 1990s (32.0 kcal/mol; 133.9 kJ/mol) (Shock and Helgeson 1990) was considerably higher than the value recently calculated by Sadowsky et al. (1.7 kcal/mol; 7.1 kJ/mol) (2013).

## Conclusions

**Fig. 7:**
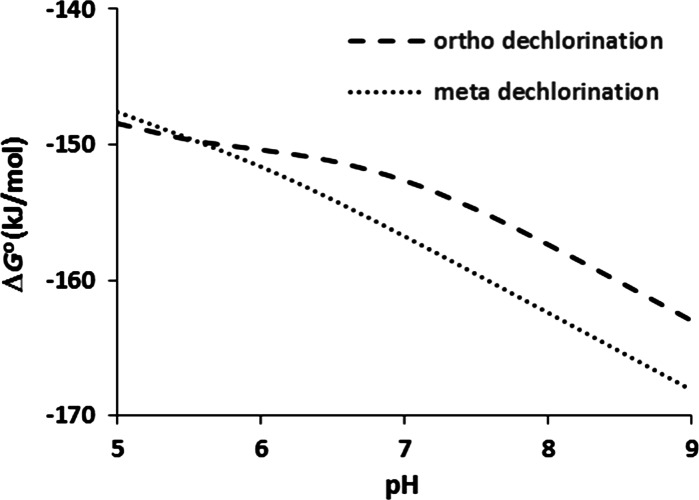
Effect of pH on the change in Gibbs free energy for the reductive dechlorination of 2,3,5,6-tetrachlorophenol to 2,3,5- and 2,3,6-trichlorophenol

The present comprehensive state-of-the-art dataset of enthalpy and Gibbs free energy of formation values of all chlorinated and brominated phenols makes it possible to calculate change in Gibbs free energy values and redox potentials for reductive dehalogenation of halogenated phenols in the aqueous phase at temperatures other than the standard temperature of 298.15 K, something that was not possible with the previously published dataset, which lacked the required enthalpy values. Other improvements include the incorporation of brominated phenols in the data set, and data for the speciation of halogenated phenols at pH 7. The effect of pH on speciation noticed above may affect which dechlorination reaction is energetically most favorable. Figure 7 shows an example where dechlorination of 2,3,5,6-tetrachlorophenol at the *ortho* position is more favorable than dechlorination at the *meta* position at pH 5 but not at pH 7.

 The data presented here illustrate that halogenated aromatics are excellent electron acceptors: the carbon-halogen bond represents a considerable source of energy. Developing technologies to harness the energy involved seems a worthwhile challenge, for example in microbial fuel cells (Huang et al. 2012). Microorganisms per se have already developed this ability (Leys et al. 2013). The classical example of microbial energy generation by organohalide respiration was with 3-chlorobenzoate as electron acceptor (Dolfing and Tiedje 1987; Dolfing 1990; Mohn and Tiedje 1990). Since then a wide variety of organohalide respiring bacteria have been identified, including organisms that can grow with halogenated benzenes, benzoates and phenols as electron acceptor (Hug et al. 2013). The present data can be used to calculate the amount of energy that is potentially available to these organisms under in situ conditions. Another potential use of the present data-set is in evaluating the dehalogenation pattern of polyhalogenated aromatics. It has been observed for various classes of halogenated compounds, including chlorophenols, that the change in Gibbs free energy values can be used to rationalize dechlorination patterns, with the energetically most favorable reactions the most likely to occur (Dolfing and Harrison 1993; Dolfing 1995, 2003), although here some restrictions apply: microorganisms and their metabolic machinery do not necessarily follow the thermodynamically predicted pathways, steric and other chemical factors may also play a role (Dolfing 2003). A case in point is the often demonstrated preferred microbial *ortho*-dehalogenation of chlorophenols (e.g. Adrian et al. 2003; Utkin et al. 1995), which is contrary to what would be expected if the organisms would preferentially use the thermodynamically most favorable pathway. Thus dehalogenation of chlorophenols by dehalogenases in *Dehalococcoides* strain DCB1 and 195 and *Desulfitobacterium dehalogenans* JW/IU-DC1 is under kinetic control, in contrast to dehalogenation of chlorophenols by vitamin B_12s_ which appears to be under thermodynamic control. The latter conclusion was drawn in 1995 based on the thermodynamic data available at that time (Dolfing 1995) and still holds when the data presented in Table 4 are used for the evaluation.

## Electronic supplementary material

Below is the link to the electronic supplementary material.

Supplementary material 1 (DOC 172 kb)
