# Supplementary material for: The Gibbs free energy of formation of halogenated benzenes, benzoates and phenols and their potential role as electron acceptors in anaerobic environments
Source: Biodegradation. 2014 Sep 18;26(1):15–27. doi: 10.1007/s10532-014-9710-5 (PMC4305373; doi:10.1007/s10532-014-9710-5)
Supplement: Supplementary file 1 — Supplementary material 1 (DOC 172 kb) [file 10532_2014_9710_MOESM1_ESM.doc]

**Table S1** Thermodynamic data for chlorophenols under standard conditions (25 ^o^C; 100 kPa in the gas phase or 1M in aqueous solution) in kJ/mol.

|  | Δ*H*_f_^o^_gas_ | Δ*G*_f_^o^_gas_ | Δ*H*_f_^o^_aq_ | Δ*G*_f_^o^_aq_ |
| --- | --- | --- | --- | --- |
| phenol | -96.40 | -51.20 | -118.00 | -72.80 |
| 2-chlorophenol | -132.80 | -83.66 | -143.89 | -95.19 |
| 3-chlorophenol | -126.30 | -77.77 | -148.44 | -99.85 |
| 4-chlorophenol | -124.20 | -75.74 | -147.01 | -98.63 |
| 5-chlorophenol | -126.10 | -77.52 | -148.31 | -99.84 |
| 6-chlorophenol | -119.50 | -70.81 | -144.28 | -95.12 |
| 2,3-dichlorophenol | -154.70 | -101.73 | -164.53 | -111.55 |
| 2,4-dichlorophenol | -156.60 | -104.17 | -167.54 | -115.63 |
| 2,5-dichlorophenol | -162.20 | -109.70 | -173.01 | -120.72 |
| 2,6-dichlorophenol | -152.60 | -99.88 | -164.71 | -112.76 |
| 3,4-dichlorophenol | -145.90 | -93.72 | -168.31 | -116.28 |
| 3,5-dichlorophenol | -151.80 | -99.88 | -173.03 | -121.15 |
| 3,6-dichlorophenol | -149.10 | -97.08 | -173.10 | -120.91 |
| 4,5-dichlorophenol | -145.60 | -93.40 | -168.09 | -115.43 |
| 4,6-dichlorophenol | -143.40 | -91.43 | -168.11 | -116.26 |
| 5,6-dichlorophenol | -141.40 | -88.59 | -165.13 | -112.39 |
| 2,3,4-trichlorophenol | -174.80 | -118.12 | -183.98 | -127.37 |
| 2,3,5-trichlorophenol | -179.60 | -123.31 | -188.27 | -132.16 |
| 2,3,6-trichlorophenol | -173.90 | -117.39 | -184.41 | -131.01 |
| 2,4,5-trichlorophenol | -177.30 | -121.12 | -186.71 | -130.20 |
| 2,4,6-trichlorophenol | -180.50 | -124.48 | -191.25 | -136.49 |
| 2,5,6-trichlorophenol | -173.53 | -117.02 | -184.42 | -128.00 |
| 3,4,5-trichlorophenol | -167.20 | -111.22 | -188.07 | -131.95 |
| 3,4,6-trichlorophenol | -164.60 | -108.96 | -187.55 | -131.77 |
| 3,5,6-trichlorophenol | -166.10 | -110.22 | -188.47 | -132.34 |
| 4,5,6-trichlorophenol | -161.20 | -104.99 | -184.34 | -128.03 |
| 2,3,4,5-tetrachlorophenol | -191.70 | -131.37 | -198.57 | -138.30 |
| 2,3,4,6-tetrachlorophenol | -192.40 | -132.17 | -200.45 | -140.03 |
| 2,3,5,6-tetrachlorophenol | -193.80 | -133.51 | -201.44 | -142.19 |
| 2,4,5,6-tetrachlorophenol | -191.60 | -131.34 | -200.16 | -139.45 |
| 3,4,5,6-tetrachlorophenol | -178.50 | -118.46 | -199.57 | -139.38 |
| pentachlorophenol | -202.50 | -137.94 | -207.85 | -144.34 |

**Table S2** Thermodynamic data for bromophenols under standard conditions (25 ^o^C; 100 kPa in the gas phase or 1M in aqueous solution) in kJ/mol.

|  | Δ*H*_f_^o^_gas_ | Δ*G*_f_^o^_gas_ | Δ*H*_f_^o^_aq_ | Δ*G*_f_^o^_aq_ |
| --- | --- | --- | --- | --- |
| phenol | -96.40 | -51.20 | -118.00 | -72.80 |
| 2-bromophenol | -79.90 | -45.09 | -90.00 | -55.00 |
| 3-bromophenol | -72.90 | -38.60 | -97.00 | -62.88 |
| 4-bromophenol | -71.00 | -36.96 | -96.03 | -62.02 |
| 5-bromophenol | -72.80 | -38.61 | -97.40 | -63.02 |
| 6-bromophenol | -67.00 | -32.71 | -91.80 | -57.33 |
| 2,3-dibromophenol | -48.70 | -24.32 | -59.53 | -35.20 |
| 2,4-dibromophenol | -52.50 | -28.74 | -64.04 | -41.00 |
| 2,5-dibromophenol | -53.70 | -29.94 | -65.14 | -41.40 |
| 2,6-dibromophenol | -49.00 | -24.92 | -60.57 | -37.60 |
| 3,4-dibromophenol | -39.40 | -15.78 | -65.57 | -41.95 |
| 3,5-dibromophenol | -46.70 | -23.50 | -72.06 | -48.95 |
| 3,6-dibromophenol | -41.10 | -17.82 | -67.34 | -43.34 |
| 4,5-dibromophenol | -39.10 | -15.47 | -65.72 | -41.70 |
| 4,6-dibromophenol | -39.80 | -16.48 | -66.90 | -43.64 |
| 5,6-dibromophenol | -35.00 | -11.05 | -62.03 | -37.87 |
| 2,3,4-tribromophenol | -13.30 | 0.10 | -42.48 | -29.70 |
| 2,3,5-tribromophenol | -21.20 | -7.72 | -32.01 | -18.45 |
| 2,3,6-tribromophenol | -16.30 | -2.70 | -27.88 | -15.44 |
| 2,4,5-tribromophenol | -18.90 | -5.58 | -30.74 | -17.58 |
| 2,4,6-tribromophenol | -19.80 | -6.87 | -30.90 | -19.58 |
| 2,5,6-tribromophenol | -15.40 | -1.79 | -15.40 | -13.88 |
| 3,4,5-tribromophenol | -4.70 | 8.53 | -31.97 | -18.95 |
| 3,4,6-tribromophenol | -6.60 | 6.26 | -33.98 | -21.55 |
| 3,5,6-tribromophenol | -7.70 | 5.24 | -34.58 | -21.20 |
| 4,5,6-tribromophenol | 0.90 | 14.31 | -28.28 | -22.89 |
| 2,3,4,5-tetrabromophenol | 23.40 | 26.83 | 12.23 | 15.89 |
| 2,3,4,6-tetrabromophenol | 20.50 | 23.69 | 9.67 | 12.70 |
| 2,3,5,6-tetrabromophenol | 18.70 | 21.93 | 8.11 | 9.25 |
| 2,4,5,6-tetrabromophenol | 21.60 | 24.65 | 9.90 | 7.30 |
| 3,4,5,6-tetrabromophenol | 36.60 | 39.57 | 8.18 | 11.96 |
| pentabromophenol | 65.60 | 59.05 | 54.80 | 45.71 |

**Table S3** Effect of p*K_a_* on speciation and Δ*G*_f_^o^_aq_ at pH 7 for bromophenols^a^

| |  |  | Δ*G*_f_^o^_aq_ | p*K_a_* | α ^b^ | Δ*G*_f_^o^*'*_aq_ ^c^ | | --- | --- | --- | --- | --- | --- | | phenol |  | -72.8 | 10.00 | 1.00 | -72.8 | | 2-bromophenol |  | -57.3 | 8.60 | 0.98 | -57.4 | | 3-bromophenol |  | -63.0 | 9.07 | 0.99 | -63.0 | | 4-bromophenol |  | -62.0 | 9.21 | 0.99 | -62.0 | | 2,3-dibromophenol |  | -37.9 | 8.13 | 0.93 | -38.0 | | 2,4-dibromophenol |  | -43.6 | 7.98 | 0.91 | -43.9 | | 2,5-dibromophenol |  | -43.3 | 7.53 | 0.77 | -44.0 | | 2,6-dibromophenol |  | -37.6 | 6.62 | 0.29 | -40.6 | | 3,4-dibromophenol |  | -42.0 | 8.68 | 0.98 | -42.0 | | 3,5-dibromophenol |  | -49.0 | 8.05 | 0.92 | -49.2 | | 2,3,4-tribromophenol |  | -29.7 | 7.61 | 0.80 | -30.2 | | 2,3,5-tribromophenol |  | -21.2 | 7.04 | 0.52 | -22.8 | | 2,3,6-tribromophenol |  | -15.4 | 6.14 | 0.12 | -20.7 | | 2,4,5-tribromophenol |  | -21.6 | 7.11 | 0.56 | -23.0 | | 2,4,6-tribromophenol |  | -19.6 | 6.05 | 0.10 | -25.3 | | 3,4,5-tribromophenol |  | -19.0 | 7.52 | 0.77 | -19.6 | | 2,3,4,5-tetrabromophenol |  | 12.0 | 6.79 | 0.38 | 9.6 | | 2,3,4,6-tetrabromophenol |  | 7.3 | 5.84 | 0.06 | 0.5 | | 2,3,5,6-tetrabromophenol |  | 9.3 | 6.14 | 0.12 | 4.0 | | pentabromophenol |  | 45.7 | 6.22 | 0.14 | 40.9 | |  |  |  |  |  |
| --- | --- | --- | --- | --- | --- | --- | --- | --- | --- | --- | --- | --- | --- | --- | --- | --- | --- | --- | --- | --- | --- | --- | --- | --- | --- | --- | --- | --- | --- | --- | --- | --- | --- | --- | --- | --- | --- | --- | --- | --- | --- | --- | --- | --- | --- | --- | --- | --- | --- | --- | --- | --- | --- | --- | --- | --- | --- | --- | --- | --- | --- | --- | --- | --- | --- | --- | --- | --- | --- | --- | --- | --- | --- | --- | --- | --- | --- | --- | --- | --- | --- | --- | --- | --- | --- | --- | --- | --- | --- | --- | --- | --- | --- | --- | --- | --- | --- | --- | --- | --- | --- | --- | --- | --- | --- | --- | --- | --- | --- | --- | --- | --- | --- | --- | --- | --- | --- | --- | --- | --- | --- | --- | --- | --- | --- | --- | --- | --- | --- | --- | --- |

^a^ p*K_a_* values are taken from Han and Tao (2006); Δ*G*_f_^o^_aq_, and Δ*G*_f_^o^*'*_aq_ are in kJ mol^-1^

^b^ α is the fraction of brominated phenol present as bromophenol; the fraction present as bromophenolate is 1-α

^c^ Δ*G*_f_^o^*'*_aq_ : Δ*G*_f_^o^_aq_ at pH 7

**Table S4** Gibbs free energy values and redox potentials for the reductive dechlorination of bromophenols with H_2_ _gas_ as electron donor^a^

|  |  |  |  |  |  |
| --- | --- | --- | --- | --- | --- |
| substrate | product | Δ*G*^o^ | Δ*G*^o^*'* | *E*^o^ | *E*^o^' |
| pentabromophenol | 2,3,4,5-tetrabromophenol | -137.8 | -175.3 | 714 | 494 |
|  | 2,3,4,6-tetrabromophenol | -142.4 | -184.3 | 738 | 541 |
|  | 2,3,5,6-tetrabromophenol | -140.5 | -180.8 | 728 | 523 |
| 2,3,4,5-tetrabromophenol | 2,3,4-tribromophenol | -145.7 | -183.8 | 755 | 538 |
|  | 3,4,5-tribromophenol | -134.9 | -173.1 | 699 | 483 |
|  | 2,4,5-tribromophenol | -137.5 | -176.5 | 712 | 501 |
|  | 2,3,5-tribromophenol | -137.2 | -176.3 | 711 | 500 |
| 2,3,4,6-tetrabromophenol | 2,3,6-tribromophenol | -126.7 | -165.2 | 657 | 442 |
|  | 2,4,6-tribromophenol | -130.9 | -169.7 | 678 | 466 |
|  | 2,3,4-tribromophenol | -141.0 | -174.7 | 731 | 491 |
|  | 2,4,5-tribromophenol | -132.9 | -167.5 | 688 | 454 |
| 2,3,5,6-tetrabromophenol | 2,3,5-tribromophenol | -134.5 | -170.8 | 697 | 471 |
|  | 2,3,6-tribromophenol | -128.7 | -168.7 | 667 | 460 |
| 2,3,4-tribromophenol | 2,3-dibromophenol | -112.2 | -151.8 | 581 | 372 |
|  | 2,4-dibromophenol | -117.9 | -157.6 | 611 | 403 |
|  | 3,4-dibromophenol | -116.3 | -155.7 | 602 | 393 |
| 2,4,5-tribromophenol | 2,4-dibromophenol | -126.1 | -164.9 | 653 | 440 |
|  | 2,5-dibromophenol | -125.8 | -165.0 | 652 | 441 |
|  | 3,4-dibromophenol | -124.4 | -163.0 | 645 | 431 |
| 2,4,6-tribromophenol | 2,4-dibromophenol | -128.1 | -162.6 | 664 | 428 |
|  | 2,6-dibromophenol | -122.0 | -159.3 | 632 | 412 |
| 2,3,5-tribromophenol | 2,3-dibromophenol | -120.7 | -159.2 | 625 | 411 |
|  | 3,5-dibromophenol | -131.8 | -170.3 | 683 | 469 |
|  | 2,5-dibromophenol | -126.1 | -165.1 | 654 | 442 |
| 2,3,6-tribromophenol | 2,3-dibromophenol | -126.4 | -161.3 | 655 | 422 |
|  | 2,6-dibromophenol | -126.2 | -163.9 | 654 | 435 |
|  | 2,5-dibromophenol | -131.9 | -167.3 | 683 | 453 |
| 3,4,5-tribromophenol | 3,4-dibromophenol | -127.0 | -166.4 | 658 | 448 |
|  | 3,5-dibromophenol | -134.0 | -173.5 | 694 | 485 |
| 2,3-dibromophenol | 2-bromophenol | -123.5 | -163.3 | 640 | 432 |
|  | 3-bromophenol | -129.2 | -169.0 | 669 | 461 |
| 2,4-dibromophenol | 2-bromophenol | -117.7 | -157.5 | 610 | 402 |
|  | 4-bromophenol | -122.4 | -162.1 | 634 | 426 |
| 2,5-dibromophenol | 2-bromophenol | -118.0 | -157.4 | 611 | 401 |
|  | 3-bromophenol | -123.7 | -163.0 | 641 | 431 |
| 2,6-dibromophenol | 2-bromophenol | -123.7 | -160.7 | 641 | 419 |
| 3,4-dibromophenol | 3-bromophenol | -125.1 | -165.0 | 648 | 441 |
|  | 4-bromophenol | -124.1 | -164.0 | 643 | 436 |
| 3,5-dibromophenol | 3-bromophenol | -118.1 | -157.8 | 612 | 404 |
| 2-bromophenol | phenol | -119.5 | -159.4 | 619 | 412 |
| 3-bromophenol | phenol | -113.8 | -153.7 | 590 | 383 |
| 4-bromophenol | phenol | -114.8 | -154.7 | 595 | 388 |

^a^ Standard conditions are 25 ^o^C, all solutes at 1 M, H_2_ gas at 1 atm; Δ*G*^o^*'* and *E*^o^' are for pH 7. Δ*G* vales are in kJ/reaction; *E* values are in mV
